# Supplementary material for: High Expression of a Cancer Stemness-Related Gene, Chromobox 8 (CBX8), in Normal Tissue Adjacent to the Tumor (NAT) Is Associated with Poor Prognosis of Colorectal Cancer Patients
Source: Cells. 2022 Jun 6;11(11):1852. doi: 10.3390/cells11111852 (PMC9180723; doi:10.3390/cells11111852)
Supplement: Supplementary file 1 [file cells-11-01852-s001.zip › supplementary figure S1.pdf]

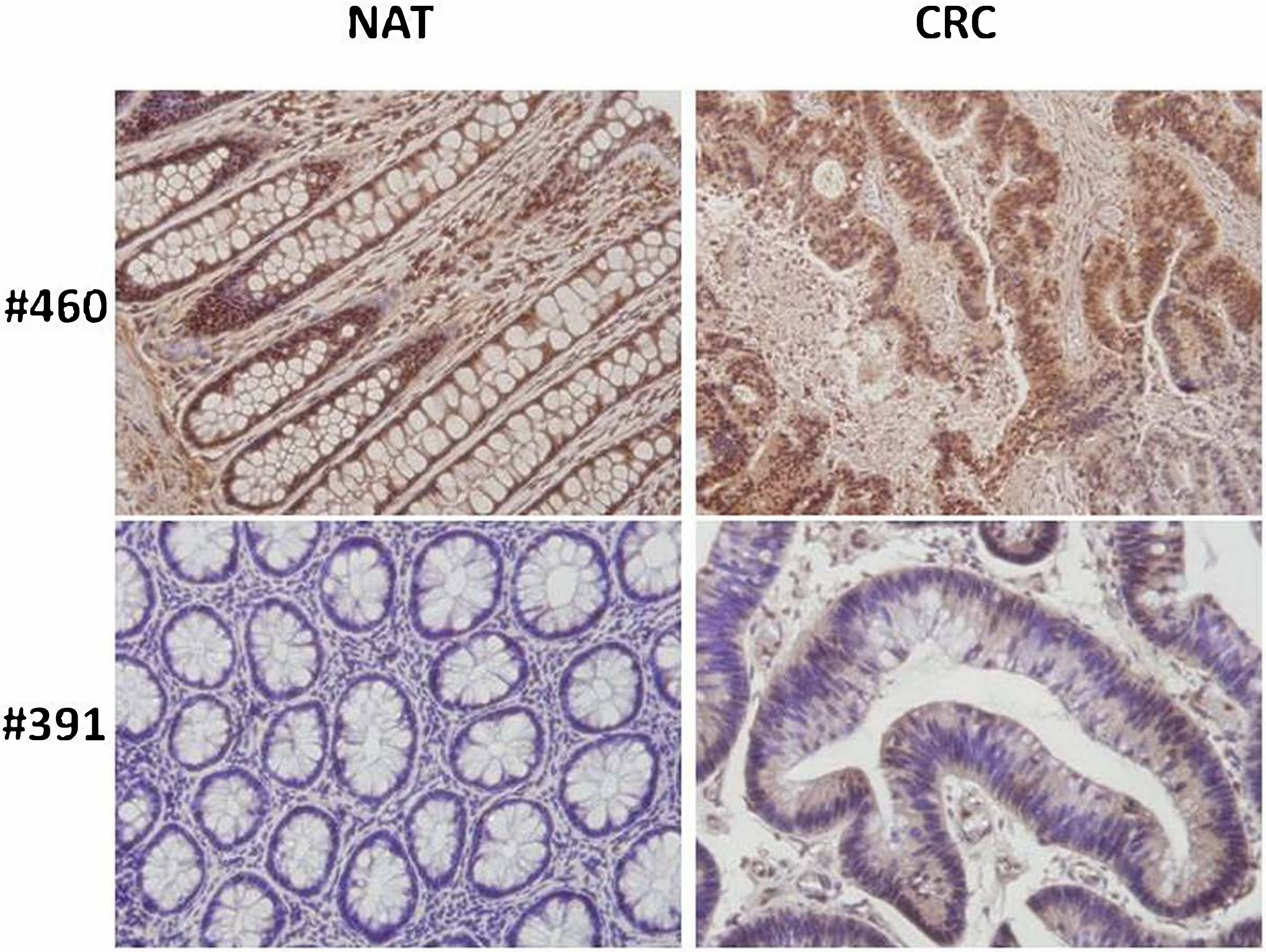

Supplementary figure S1

Representative IHC stainings of CBX8 in patients #460 and #391 showed high level of CBX8 (#460) and low CBX8 (#391) in NAT and CRC specimens (Magnification: 40X). As shown by sample #460, crypt epithelial cells and stroma cells in NAT specimens are able to express CBX8.
