# Supplementary material for: High Expression of a Cancer Stemness-Related Gene, Chromobox 8 (CBX8), in Normal Tissue Adjacent to the Tumor (NAT) Is Associated with Poor Prognosis of Colorectal Cancer Patients
Source: Cells. 2022 Jun 6;11(11):1852. doi: 10.3390/cells11111852 (PMC9180723; doi:10.3390/cells11111852)
Supplement: Supplementary file 1 [file cells-11-01852-s001.zip › supplementary figure S2.pdf]

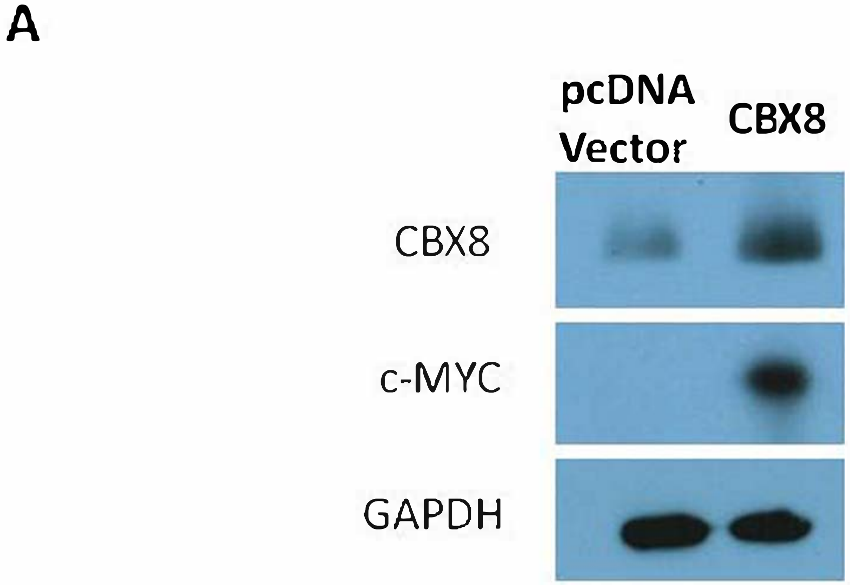

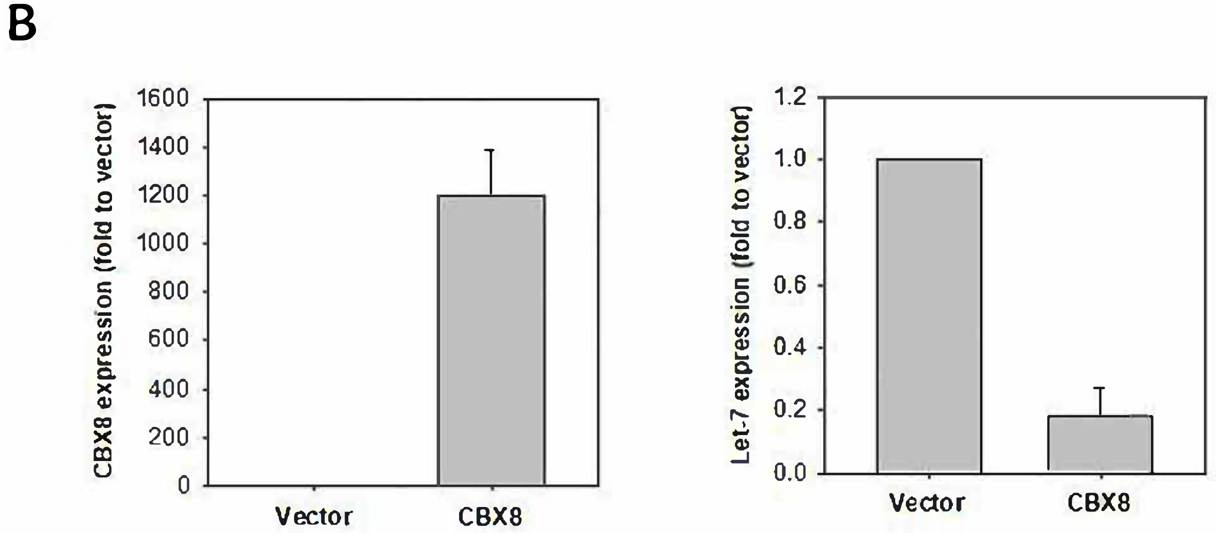

Supplementary figure S2

(A) Western blot results shows that overexpression of CBX8 in CRC cell-line HCT116 induced expression of c-MYC when compared to pcDNA vector control. GAPDH was used as internal control. (B) Quantitative PCR results show that CBX8 overexpression in HCT116 cells induced CBX8 gene level (left) and down-regulated microRNA let-7 level.
